# Association of Skeletal Muscle Radiodensity and Skeletal Muscle Index with Immunotherapy Response in Metastatic Non-Small Cell Lung Cancer

**DOI:** 10.3390/muscles4040051

**Published:** 2025-11-05

**Authors:** Yuliia Moskalenko, Viktor Kovchun, Ihor Vynnychenko, Roman Moskalenko

**Affiliations:** 1 Department of Oncology and Radiology, Sumy State University, 40022 Sumy, Ukraine; 2 Department of Pathology, Sumy State University, 40000 Sumy, Ukraine

**Keywords:** skeletal muscle index, skeletal muscle radiodensity, non-small cell lung cancer, disease control rate, survival

## Abstract

Sarcopenia and reduced skeletal muscle radiodensity have been proposed as potential biomarkers influencing the outcomes of immunotherapy in cancer patients. This retrospective study aimed to evaluate the prognostic significance of skeletal muscle index (SMI) and skeletal muscle radiodensity (SMD), assessed by means of computed tomography imaging at the L3 level, in 76 male patients with metastatic non-small cell lung cancer treated with PD-1/PD-L1 inhibitors. Patients were categorized into high and low SMI/SMD groups based on body mass index-adjusted cut-off values. Clinical outcomes included treatment response, overall survival, and immune-related adverse events. While no statistically significant differences in overall survival were observed between groups stratified by SMI or SMD, patients with higher SMD demonstrated a significantly greater disease control rate (56.22 ± 8.04 vs. 48.36 ± 10.34 HU; *p* = 0.031). Additionally, a statistically significant interaction was observed between PD-L1 expression and SMI (*p* = 0.027), indicating that muscle mass may influence the prognostic value of PD-L1. Neither SMI nor SMD were associated with immune-related adverse event incidence. Multivariate analysis identified PD-L1 expression ≥ 50% as the only independent predictor of longer overall survival (Hazard Ratio = 0.29; *p* = 0.001). In conclusion, while neither SMI nor SMD independently predicted overall survival, SMD was associated with treatment response. Notably, SMI modified the prognostic relevance of PD-L1 expression, suggesting a potential role for muscle mass in refining immunotherapy stratification.

## 1. Introduction

Sarcopenia is a syndrome characterized by a progressive decline in skeletal muscle mass, strength, and functional capacity. In cancer patients, sarcopenia is frequently associated with cancer cachexia and is considered an independent adverse prognostic factor [1]. A reduction in muscle mass negatively affects functional status, treatment tolerance, complication rates, and overall survival in patients with solid tumors, including non-small cell lung cancer (NSCLC) [2].

Although various methods exist for assessing muscle mass (such as anthropometric measurements or bioelectrical impedance analysis), computed tomography (CT) has emerged as the gold standard for the quantitative evaluation of skeletal muscle in clinical oncology, due to its high precision and suitability for retrospective analysis [3]. The skeletal muscle index (SMI) and skeletal muscle radiodensity (SMD), measured at the lumbar or thoracic vertebral levels (T10 to L5), enable objective characterization of both the quantity and quality of muscle tissue [4].

The advent of immunotherapy, particularly immune checkpoint inhibitors (ICIs), has revolutionized the treatment landscape of NSCLC. Anti-PD-1/PD-L1 agents have significantly improved outcomes in patients with advanced disease, especially those with PD-L1 expression levels ≥ 50% [5]. However, not all patients demonstrate a durable immune response, highlighting the need for novel predictive biomarkers—among which sarcopenia has recently attracted increasing attention.

Emerging evidence suggests that sarcopenia may negatively influence the effectiveness of ICIs. Meta-analytical data encompassing over 1800 lung cancer patients have demonstrated that sarcopenia is associated with poorer overall survival and increased disease progression [6]. Retrospective analyses further support a link between low skeletal muscle mass and reduced disease control rates in patients undergoing immunotherapy [7], as well as a significantly decreased likelihood of achieving an objective response and a higher risk of progression [8].

In addition to reduced efficacy, sarcopenia has also been associated with a higher incidence of grade ≥ 3 toxicities in patients receiving ICIs [9]. Other studies have corroborated these findings, showing that individuals with sarcopenia or cachexia experience worse overall survival and lower disease control rates compared to patients with preserved muscle mass [10].

However, not all investigations have confirmed these associations. Some studies have reported no statistically significant differences in survival based on sarcopenia status [11], and the impact of SMI on treatment outcomes appears to be limited, particularly among patients receiving combination chemoimmunotherapy [12]. Moreover, in certain cohorts, sarcopenia has not emerged as a significant prognostic factor, while PD-L1 expression has proven to be a more robust predictor of clinical outcomes [13]. Other analyses suggest that broader indicators of nutritional status may offer greater predictive value than SMI alone [14,15].

Given these conflicting findings, it remains unclear whether sarcopenia is an independent predictor of immunotherapy response or merely a surrogate marker of overall clinical condition. This underscores the need for further investigation, taking into account not only SMI but also parameters reflecting muscle quality (such as SMD), tumor histological subtype, body mass index (BMI), and PD-L1 expression.

The aim of the study was to determine the impact of SMI and SMD, as assessed by means of CT imaging, on the clinical efficacy of immunotherapy in patients with metastatic NSCLC.

## 2. Materials and Methods

### 2.1. Study Design and Participants

This was a retrospective, single-center study conducted at the Sumy Regional Clinical Oncology Center (Ukraine). It included a cohort of patients with metastatic NSCLC who received immunotherapy with PD-1/PD-L1 inhibitors as either first- or second-line treatment between 2016 and 2024.

Eligible participants were male patients with a confirmed diagnosis of metastatic NSCLC who were treated with pembrolizumab or atezolizumab—either as monotherapy or in combination with chemotherapy. A prerequisite for inclusion was the availability of an abdominal CT scan performed no later than 30 days prior to the initiation of immunotherapy, which enabled assessment of skeletal muscle mass and radiodensity. Complete clinical data on treatment response, survival outcomes, and irAEs were also required.

Exclusion criteria included absence of a baseline CT scan of appropriate quality or within the defined timeframe, incomplete or incorrectly documented clinical records, and the presence of comorbid conditions or physical states that could significantly affect muscle structure or radiodensity (e.g., severe chronic liver failure, lumbar spine trauma or surgery). Female patients were excluded due to insufficient sample size for statistically valid sex-based stratification.

### 2.2. Assessment of Treatment Response and Clinical Variables

Treatment response was assessed according to iRECIST criteria, based on the best overall response [16]. Objective response rate (ORR) was defined as the proportion of patients who achieved an objective response, i.e., complete or partial response. Disease control rate (DCR) was defined as the proportion of patients who achieved disease control, i.e., complete response, partial response, or stable disease. Overall survival was calculated from the initiation of immunotherapy to death from any cause. Progression-free survival was defined as the time from initiation of immunotherapy to documented disease progression or death, whichever occurred first. Adverse events were recorded and graded according to CTCAE v5.0 (U.S. National Cancer Institute), and only those considered potentially immune-related were included in the analysis.

In addition to muscle-related metrics, the study considered several clinicopathologic variables, including: age (<60 vs. ≥60 years), BMI (≤24.9 vs. >24.9), smoking status (current/former vs. never), tumor histology (adenocarcinoma vs. squamous cell carcinoma), PD-L1 expression level (1–49% vs. ≥50%), treatment line (first vs. second), and treatment regimen (immunotherapy vs. chemo-immunotherapy). These data were extracted from primary medical records. PD-L1 expression was categorized as 1–49% and ≥50%, in line with international treatment guidelines. These thresholds are recommended as clinically significant by both the National Comprehensive Cancer Network and the European Society for Medical Oncology for selecting treatment strategies in patients with advanced non-small cell lung cancer.

### 2.3. Assessment of Muscle Mass and Radiodensity

**Figure 1:**
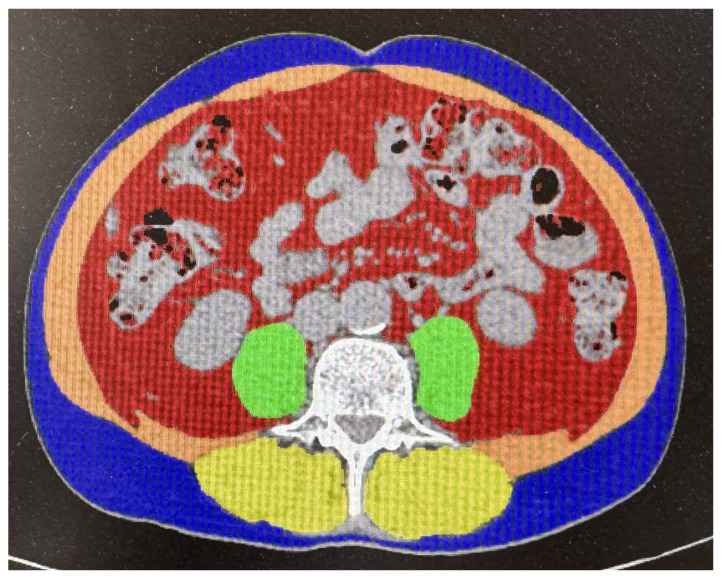
CT evaluation of the cross-sectional area of lumbar skeletal muscles at the L3 level using Synapse 3D. Muscles are highlighted in yellow and orange.

Only patients who underwent abdominal CT scanning within 30 days before the start of immunotherapy were eligible for analysis. Muscle mass was assessed on axial CT images at the level of the third lumbar vertebra (L3), ensuring clear visualization of both transverse processes. Images were analyzed using Synapse 3D (Fujifilm 4.4, Tokyo, Japan; server-side thin-client platform with >50 clinical 3D modules). All measurements were performed by a board-certified oncologic radiologist with over 10 years of experience, blinded to clinical outcomes (Figure 1).

To eliminate the effect of contrast medium, only scans acquired during the basal or early arterial phase were used [17]. Skeletal muscle was identified using a Hounsfield unit (HU) range from −29 to +150. The SMI was calculated as the cross-sectional muscle area (cm^2^) divided by the square of the patient’s height (m^2^). The analysis included psoas, paraspinal, and abdominal wall muscles, excluding any visceral components. SMD was automatically determined as the mean HU value across the entire muscle area at the L3 level.

Predefined cut-off values for SMI and SMD derived from pre-ICI studies [18] were not applied. Instead, thresholds were determined based on the characteristics of the study population, stratified by BMI (<24.9 vs. ≥25.0). Accordingly, patients were categorized as having either low (indicative of sarcopenia) or high SMI and low or high SMD.

### 2.4. Statistical Analysis

All statistical analyses were performed using Stata version 19.5 (StataCorp, TX, USA; www.stata.com; accessed on 23 August 2025). Categorical variables were presented as absolute numbers and percentages, and continuous variables as mean ± standard deviation or median with interquartile range (IQR), depending on distribution. Associations between SMI, SMD, and clinical outcomes (ORR, DCR, OS, and irAEs) were evaluated using the chi-square test or Fisher’s exact test, as appropriate. The Mann–Whitney U test was used to compare continuous variables (e.g., median SMI or SMD between responders and non-responders).

The prognostic value and optimal cut-off points for SMI and SMD were assessed via receiver operating characteristic (ROC) curve analysis, with calculation of the area under the curve (AUC). Survival analysis was conducted using the Kaplan–Meier method with comparisons made by the log-rank test. Multivariable Cox proportional hazards regression was used to identify independent predictors of overall survival, with results reported as hazard ratios (HR) and 95% confidence intervals (CI). A *p*-value of <0.05 was considered statistically significant. Interaction analyses were additionally performed to assess whether the prognostic effects of SMI and SMD on overall survival were modified by clinicopathological variables. This was done by including multiplicative interaction terms in the multivariate Cox regression models for age, smoking status, histology, BMI, PD-L1 expression, and treatment regimen.

## 3. Results

### 3.1. General Characteristics of the Study Cohort

A total of 76 male patients with metastatic NSCLC were included in the study. The majority were smokers (88.2%). The mean age of the patients was 61.4 ± 7.05 years. Histologically, squamous cell carcinoma predominated (59.2%) compared to adenocarcinoma (40.8%). PD-L1 expression ≥ 50% was observed in 38.2% of cases, while 61.8% had expression levels ranging from 1% to 49%. Based on BMI, patients were nearly equally distributed between those with BMI < 24.9 (52.6%) and ≥25.0 (47.4%). The mean cross-sectional area of lumbar skeletal muscles in the cohort was 171.0 ± 44.9 cm^2^.

The mean SMI in the cohort was 58.0 ± 15.46 cm^2^/m^2^. ROC curve analysis showed that the SMI had adequate sensitivity and specificity (AUC = 0.6577, 95% CI: 0.48316–0.79372 for BMI < 24.9; AUC = 0.7794, 95% CI: 0.60848–0.89885 for BMI ≥ 25.0). The SMI threshold values for stratifying patients into low and high SMI subgroups were determined to be 39.0 cm^2^/m^2^ for BMI < 24.9 and 60.0 cm^2^/m^2^ for BMI ≥ 25.0. Low SMI, indicative of sarcopenia, was identified in 26.3% of patients.

The mean SMD among male patients with NSCLC was 49.29 ± 10.37 (HU), with low radiodensity observed in 18.4% of cases. Similar to SMI, the SMD thresholds were calculated using ROC analysis, although this parameter demonstrated lower sensitivity and specificity (AUC = 0.3649, 95% CI: 0.22726–0.54199 for BMI < 24.9; AUC = 0.5147, 95% CI: 0.35486–0.69595 for BMI ≥ 25.0). The cut-off values for SMD were established at 40.0 HU for BMI < 24.9 and 53.0 HU for BMI ≥ 25.0.

Regarding treatment response, a partial response was observed in 44.7% of patients, disease stabilization in 35.5%, complete response in 8.0%, and disease progression in 11.8% (Table 1).

### 3.2. Overall Survival and Progression-Free Survival According to SMI and SMD

**Figure 2:**
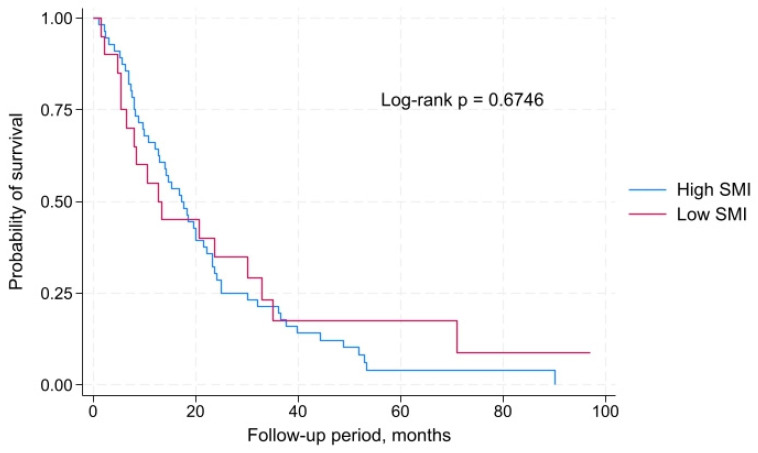
Overall survival of male patients with metastatic non-small cell lung cancer stratified by skeletal muscle index (low vs. high).

Comparison of overall survival between patients with low and high SMI revealed no statistically significant differences. The median overall survival in patients with high SMI was 17.2 months (IQR: 8.0–25.1 months), whereas in those with low SMI it was 12.6 months (IQR: 5.5–32.9 months). Kaplan–Meier survival curve analysis showed no significant difference between the groups according to the log-rank test: χ^2^(1) = 0.18, *p* = 0.6746. Thus, in this patient cohort, SMI was not associated with overall survival (Figure 2).

**Figure 3:**
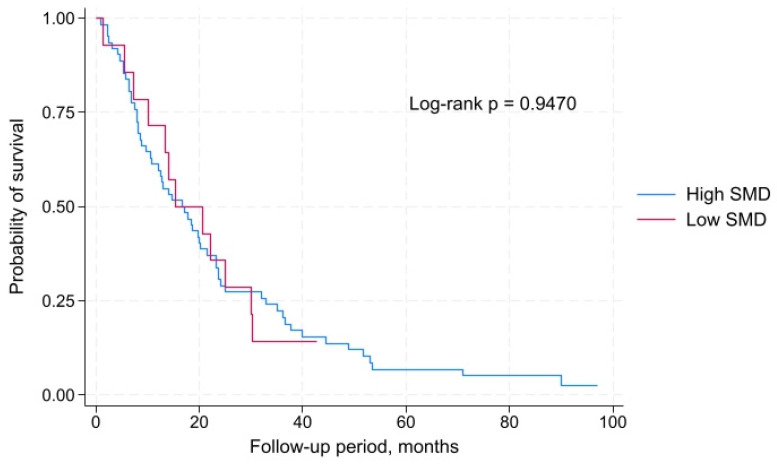
Overall survival of male patients with metastatic non-small cell lung cancer stratified by skeletal muscle radiodensity (low vs. high).

Analysis of overall survival according to SMD also revealed no statistically significant differences between groups. Patients with high SMD had a median overall survival of 16.8 months (IQR: 7.9–32.9 months), while those with low SMD had a median overall survival of 15.3 months (IQR: 10.0–30.1 months). According to the log-rank test, the survival curves did not differ significantly: χ^2^(1) = 0.00, *p* = 0.9470. Therefore, SMD was not associated with overall survival in this patient cohort (Figure 3).

In the analysis of progression-free survival, no significant differences were observed between low and high SMI groups (median progression-free survival: 8.37 vs. 5.5 months, respectively; log-rank *p* = 0.3672; Supplementary Figure S1). Similarly, there was no significant difference in progression-free survival between patients with low and high SMD (median progression-free survival: 6.77 vs. 11.17 months, respectively; log-rank *p* = 0.6049; Supplementary Figure S2).

### 3.3. Association of SMI and SMD with Treatment Response

An objective response to treatment was achieved in 40 out of 76 patients (52.6%), while disease control was observed in 67 out of 76 patients (88.2%). Patients who achieved an objective treatment response had a higher mean SMI (60.52 ± 17.49 cm^2^/m^2^) compared to those without response (55.18 ± 12.50 cm^2^/m^2^). Similarly, patients who attained disease control demonstrated higher SMI values (58.66 ± 15.14 cm^2^/m^2^) than those who did not (53.00 ± 17.84 cm^2^/m^2^). However, the differences in SMI between groups were not statistically significant either for objective response (*p* = 0.460) or for disease control (*p* = 0.730), indicating a non-significant trend rather than a definitive effect.

As for SMD, mean values were comparable between patients who responded to treatment (48.85 ± 10.02 HU) and those who did not (49.78 ± 10.88 HU). However, SMD was higher among patients who achieved disease control (56.22 ± 8.04 HU) compared to those who did not (48.36 ± 10.34 HU). Although the difference in SMD for objective response was not significant (*p* = 0.532), a statistically significant difference was observed for disease control (*p* = 0.031), suggesting that reduced muscle radiodensity may negatively impact immunotherapy efficacy (Table 2).

These findings complement previous observations by confirming a trend toward better clinical response in patients with higher SMI, and emphasize the prognostic significance of SMD in predicting disease control rate.

### 3.4. Association SMI, SMD, and Immune-Related Adverse Events (irAEs)

In our study, irAEs of any grade were reported in 20 out of 76 patients (26.3%). These included hyperthyroidism (*n* = 4), hypothyroidism (*n* = 1), pruritus (*n* = 1), pneumonitis (*n* = 2), colitis (*n* = 2), hepatitis (*n* = 2), bullous pemphigoid (*n* = 1), arthralgia (*n* = 1), myalgia (*n* = 1), onycholysis (*n* = 1), rash (*n* = 1), infusion reactions (*n* = 2), and aseptic bone necrosis (*n* = 1).

Among patients with sarcopenia, 3 out of 20 (15.0%) experienced irAEs, compared to 17 out of 56 (30.4%) among those without sarcopenia. Although irAEs occurred more frequently in non-sarcopenic patients, this difference did not reach statistical significance (*p* = 0.243).

When stratified by SMD, irAEs were observed in 5 out of 14 patients (35.7%) with low SMD and in 15 out of 62 patients (24.2%) with high SMD. This difference was likewise not statistically significant (*p* = 0.377).

Thus, in our cohort of male patients with metastatic NSCLC, the development of irAEs was not significantly associated with either sarcopenia or decreased muscle radiodensity. Despite certain distributional trends, no statistically significant associations were found, which may be due to the limited sample size. Larger prospective studies are warranted to further elucidate these findings.

### 3.5. Independent Predictors of Overall Survival and Interaction Analysis

To identify independent predictors of overall survival and assess possible effect modification by body composition, we performed multivariate Cox regression analysis incorporating interaction terms between SMI/SMD and key clinicopathological variables (Table 3).

Among the evaluated parameters, only PD-L1 expression ≥50% emerged as a statistically significant factor associated with improved survival (HR = 0.29; 95% CI: 0.15–0.55; *p* = 0.001). This indicates that patients with high PD-L1 expression had approximately a 3.5-fold lower risk of death compared to those with PD-L1 expression ranging from 1% to 49%. Other variables, including age, smoking status, histological subtype, BMI, treatment regimen, SMI, and SMD, did not demonstrate a significant impact on overall survival in this cohort (*p* > 0.05 for all).

These findings suggest that among the clinical and morphometric parameters analyzed, only high PD-L1 expression served as an independent predictor of longer overall survival. This underscores the pivotal prognostic value of immune response biomarkers compared to conventional clinicopathological characteristics or body composition metrics. At the same time, the absence of a significant association between SMI or SMD and survival in our cohort highlights the need for larger-scale studies to clarify their potential role in predicting immunotherapy outcomes.

Additionally, to assess whether the prognostic value of body composition measures depended on these variables, we conducted interaction analyses between SMI or SMD and age, smoking status, histology, BMI, PD-L1 expression, and treatment regimen. The interaction between PD-L1 expression and SMI was statistically significant (*p* for interaction = 0.027), suggesting that the prognostic impact of PD-L1 may differ depending on skeletal muscle mass. In contrast, no significant interaction was found between PD-L1 and SMD (*p* = 0.105), nor with other assessed variables (all *p* > 0.1), indicating that the effects of SMI and SMD on survival were largely independent of these clinicopathological features.

## 4. Discussion

Our findings suggest that SMD may serve as a useful predictor of immunotherapy efficacy in male patients with metastatic NSCLC. Although SMI did not show a statistically significant association with overall survival, there was a non-significant trend toward improved survival among patients with higher SMI values. In contrast, SMD was significantly correlated with disease control rate; patients who achieved stable disease, partial, or complete response to treatment more frequently had higher SMD values. These results indicate that the quality, rather than the quantity, of muscle tissue may be more clinically relevant in predicting immunotherapy response.

Another important observation was the statistically significant interaction between SMI and PD-L1 expression in predicting overall survival. The survival benefit associated with high PD-L1 expression (≥50%) was more pronounced in patients with preserved muscle mass, suggesting that muscle quantity may influence the prognostic effect of immune checkpoint biomarkers. This may reflect the patient’s overall immunometabolic fitness or physiological resilience to systemic therapy.

The biological mechanisms underlying these associations are likely multifactorial. Low SMI reflects diminished muscle mass, which is associated with reduced anabolic activity, nutritional deficiencies, impaired energy metabolism, and limited physiological reserve [19]. Sarcopenia is also linked to chronic systemic inflammation, T-cell exhaustion, dysbiosis, and elevated pro-inflammatory cytokine levels, which may compromise the efficacy of PD-1/PD-L1 inhibitors [20]. By contrast, higher muscle mass has been associated with improved protein turnover, better regulation of inflammatory responses, and enhanced T-cell function—factors essential for optimal immune checkpoint inhibition. It is plausible that sarcopenic patients, even with favorable PD-L1 expression, may not derive full survival benefit due to impaired immune responsiveness or catabolic states [21,22].

Reduced SMD indicates fatty infiltration of skeletal muscle (myosteatosis), which reflects a loss of structural integrity, decreased contractile capacity, heightened local inflammation, and oxidative stress [23]. These alterations may impair drug pharmacokinetics and the generation of effective anti-tumor immune responses. Thus, SMD may serve as an indirect marker of systemic metabolic and immune status [24]. These findings underscore the importance of accounting for host-related factors, such as body composition, when interpreting PD-L1 status and planning immunotherapy strategies. Future studies should explore whether interventions aimed at improving muscle health can enhance treatment outcomes in this population.

Our results are in partial agreement with previously published studies. For example, sarcopenia has been associated with significantly reduced overall and progression-free survival in NSCLC patients treated with ICIs [25]. Meta-analyses have confirmed that low SMI and SMD are associated with reduced treatment response, although the strength of this association varies depending on anatomical measurement site and patient characteristics [26]. Broader analyses across multiple malignancies have similarly demonstrated significantly lower odds of achieving objective or disease control responses, and a higher risk of mortality in patients with sarcopenia [27]. In our study, SMD—but not SMI—emerged as a more reliable indicator of disease control rather than overall survival.

Additional support comes from studies in NSCLC patients treated with nivolumab, where lower SMD was linked to significantly poorer disease control rates [7]. In contrast, other analyses, including patients receiving chemoimmunotherapy, have reported no significant associations between SMI and survival outcomes [11,12]. These findings are consistent with our data, where SMI showed only a non-significant trend toward prognostic relevance.

Further retrospective analyses suggest that although sarcopenia negatively impacts survival, high PD-L1 expression (≥50%) remains the only independent prognostic factor in multivariate models [13]. This reinforces our findings and highlights the value of a multifactorial prognostic approach in immuno-oncology.

In terms of safety, no statistically significant association was found between irAE incidence and either SMI or SMD. Patients with reduced muscle metrics did not experience increased rates of adverse events, suggesting that these parameters have limited predictive utility for treatment-related toxicity. These results are in line with previous studies reporting no effect of sarcopenia on irAE occurrence [11]. However, other investigations have observed higher toxicity rates in sarcopenic populations [9]. Discrepancies may stem from methodological differences, including irAE definitions, treatment regimens, sample size, and heterogeneity in sarcopenia assessment [28].

The clinical implication of our findings lies in the potential utility of SMD as an adjunct biomarker, especially in patients with low or ambiguous PD-L1 expression. Incorporating skeletal muscle quality assessment into baseline evaluations may enable early identification of high-risk individuals and support timely implementation of nutritional or physical rehabilitation strategies [29,30].

Several limitations should be noted. First, the retrospective design limits causal inference and may introduce selection bias. Second, the relatively small sample size—particularly the subgroups with low SMI and SMD—may have reduced statistical power, especially in multivariate or interaction analyses. Third, the study included only male patients, limiting the generalizability of findings. This was a deliberate choice based on the small number of eligible females (*n* = 16) and known sex-based differences in muscle metabolism. Lastly, functional status and inflammatory markers were not assessed, which could have provided additional context. These limitations underscore the need for large-scale, prospective, multicenter studies including both sexes and integrating biological, functional, and quality-of-life metrics to refine the prognostic value of body composition in immunotherapy for metastatic NSCLC.

## 5. Conclusions

Skeletal muscle index and skeletal muscle radiodensity were not identified as independent predictors of overall survival in male patients with metastatic non-small cell lung cancer. However, higher SMD was associated with better disease control, suggesting its predictive value in assessing immunotherapy response. Furthermore, interaction analysis revealed that the prognostic impact of PD-L1 expression on overall survival may be modulated by skeletal muscle mass, indicating that SMI could influence the clinical utility of immune checkpoint biomarkers. The frequency of immune-related adverse events was not influenced by SMI or SMD. These findings suggest that incorporating body composition assessment may improve patient stratification prior to immunotherapy, although prospective validation in larger, more diverse cohorts is necessary.

## Figures and Tables

**Table 1:** Clinicopathological and radiological characteristics of male patients with metastatic non-small cell lung cancer.

Baseline Characteristics	Total Number of Patients, *n* = 76 (100%)
Mean age (years)	61.4 ± 7.05
Smoking status: Current/former Never	67 (88.2%) 9 (11.8%)
Histology: Adenocarcinoma Squamous cell carcinoma	31 (40.8%) 45 (59.2%)
BMI: <24.9 ≥25.0	40 (52.6%) 36 (47.4%)
PD-L1 Expression: 1–49% ≥50%	57 (61.8%) 19 (38.2%)
Treatment regimen: Immunotherapy Chemoimmunotherapy	28 (36.8%) 48 (63.2%)
Line of therapy: First-line Second-line	70 (92.1%) 6 (7.9%)
Mean lumbar skeletal muscle area (cm^2^)	171.0 ± 44.9
SMI: High Low	56 (73.7%) 20 (26.3%)
Mean SMI (cm^2^/m^2^)	58.0 ± 15.46
SMD (HU): High Low	62 (81.6%) 14 (18.4%)
Mean SMD (HU)	49.29 ± 10.37
Treatment response: Complete response Stable disease Partial response Disease progression	6 (8.0%) 27 (35.5%) 34 (44.7%) 9 (11.8%)

**Table 2:** Treatment response in patients with metastatic non-small cell lung cancer according to skeletal muscle index and radiodensity.

Treatment Response		Mean SMI	Mean SMD (HU)
Objective response rate	Not achieved	55.18 ± 12.50	49.78 ± 10.88
	Achieved	60.52 ± 17.49	48.85 ± 10.02
*p*-value		0.460	0.532
Disease control rate	Not achieved	53.00 ± 17.84	48.36 ± 10.34
	Achieved	58.66 ± 15.14	56.22 ± 8.04
*p*-value		0.730	0.031

Note: The *p*-value was calculated using the Mann–Whitney U test.

**Table 3:** Multivariate Cox regression and interaction analysis of the impact of clinicopathological characteristics and body composition (SMI and SMD) on overall survival in patients with metastatic non-small cell lung cancer.

Variables	Overall Survival			*p* for Interaction	
	Hazard Ratio	95% CI	*p*	SMI	SMD
Age: <60 years ≥60 years	1 1.58	- 0.90–2.56	- 0.113	0.986	0.311
Smoking status: Never Current/former	1 0.82	- 0.39–1.74	- 0.617	0.489	0.401
Histology: Adenocarcinoma Squamous cell carcinoma	1 1.34	- 0.81–2.20	- 0.244	0.175	0.270
BMI: <24.9 ≥25.0	1 0.91	- 0.67–1.23	- 0.555	0.280	0.213
PD-L1 expression: 1–49% ≥50%	1 0.29	- 0.15–0.55	- 0.001	0.027	0.105
Regimen: Immunotherapy Chemoimmunotherapy	1 1.08	- 0.65–1.77	- 0.760	0.693	0.185
SMI: Low Hight	1 0.94	- 0.53–1.65	- 0.832	-	0.146
SMD: Hight Low	1 1.12	- 0.54–2.32	- 0.755	0.497	-

## Data Availability

The data that support the findings of this study are owned by a third party - the Sumy Regional Clinical Oncology Center (Sumy, Ukraine). Access to these data is restricted due to institutional regulations and ethical considerations. Researchers who wish to obtain the data for justified scientific purposes must first receive approval from the local ethics committee. To initiate this process, please contact the first author Yuliia Moskalenko.

## Supplementary Materials

The following supporting information can be downloaded at https://www.mdpi.com/article/10.3390/muscles4040051/s1. Figure S1: Progression-free survival of male patients with metastatic non-small cell lung cancer stratified by skeletal muscle index (low vs. high); Figure S2: Progression-free survival of male patients with metastatic non-small cell lung cancer stratified by skeletal muscle radiodensity (low vs. high).

## Abbreviations

The following abbreviations are used in this manuscript:

NSCLC	Non-small cell lung cancer
ICIs	Immune checkpoint inhibitors
SMI	Skeletal muscle index
SMD	Skeletal muscle radiodensity
ORR	Objective Response Rate
DCR	Disease Control Rate
HU	Hounsfield Units
HR	Hazard Ratio
CI	Confidence Interval
