# Supplementary material for: Association of Skeletal Muscle Radiodensity and Skeletal Muscle Index with Immunotherapy Response in Metastatic Non-Small Cell Lung Cancer
Source: Muscles. 2025 Nov 5;4(4):51. doi: 10.3390/muscles4040051 (PMC12641972; doi:10.3390/muscles4040051)
Supplement: Supplementary file 1 [file muscles-04-00051-s001.zip › Suppl_figure_signature.pdf]

Supplementary Figure S1. Progression-free survival of male patients with metastatic non-small cell lung cancer stratified by skeletal muscle index (low vs. high).

Supplementary Figure S2. Progression-free survival of male patients with metastatic non-small cell lung cancer stratified by skeletal muscle radiodensity (low vs. high).
