# Supplementary figures and images for: Association of Skeletal Muscle Radiodensity and Skeletal Muscle Index with Immunotherapy Response in Metastatic Non-Small Cell Lung Cancer
Source: Muscles. 2025 Nov 5;4(4):51. doi: 10.3390/muscles4040051 (PMC12641972; doi:10.3390/muscles4040051)

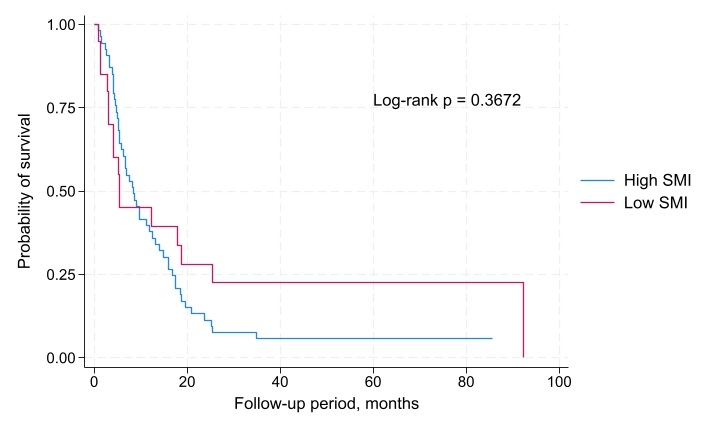

Supplement: Supplementary file 1 [file muscles-04-00051-s001.zip › Supplementary figure S1.jpg]

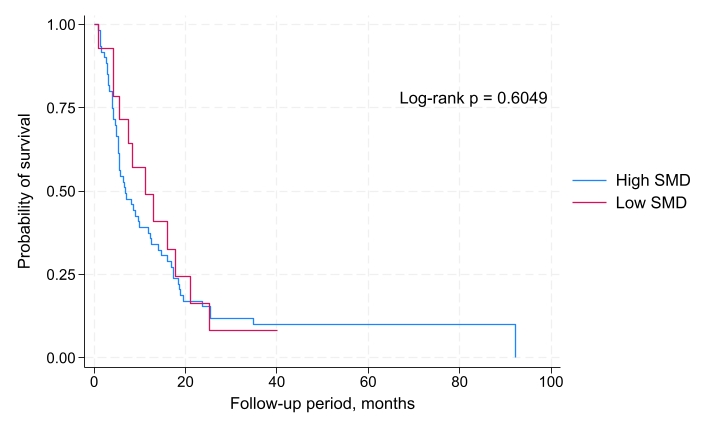

Supplement: Supplementary file 1 [file muscles-04-00051-s001.zip › Supplementary figure S2.jpg]
